# Long-Term Outcomes and Prognoses of Elderly Patients (≥65-Years-Old) With Distant Metastases From Well-Differentiated Thyroid Cancer During Radioiodine Therapy and Follow-Up

**DOI:** 10.3389/fendo.2020.588024

**Published:** 2021-02-25

**Authors:** Zhong-Ling Qiu, Chen-Tian Shen, Zhen-Kui Sun, Hong-Jun Song, Chuang Xi, Guo-Qiang Zhang, Yang Wang, Quan-Yong Luo

**Affiliations:** Department of Nuclear Medicine, Shanghai Jiao Tong University Affiliated Sixth People’s Hospital, Shanghai, China

**Keywords:** distant metastases, radioiodine therapy, elderly patients, well-differentiated thyroid cancer, follow-up

## Abstract

**Objective:**

The objective of this study was to investigate the clinicopathological characteristics, long-term outcomes, and prognostic factors of elderly patients with distant metastases at initial diagnosis from well-differentiated thyroid cancer (WDTC) during radioactive iodine (^131^I) treatment and follow-up.

**Methods:**

A retrospective review of medical records identified 183 elderly patients with DTC who underwent ^131^I treatment at our institution between 2006 and 2019.

**Results:**

In total, 57 elderly WDTC patients with distant metastases were enrolled in this study. After ^131^I treatment, 32 (56.14%) patients had ^131^I avidity and 25 (43.86%) had non-^131^I avidity; 35 (61.40%) cases were classified as radioiodine refractory (RR)-WDTC and 22 (38.60%) as non-RR-WDTC. At the end of follow-up, 25 (43.86%) patients had died and 32 (56.14%) were alive. The 5- and 10-year overall survival (OS) rates were 71.50% and 30.49%, respectively, while the 5- and 10-year disease-specific survival (DSS) rates were 76.89% and 48.71%, respectively. Multivariate analyses showed that gross extrathyroidal extension and RR-DTC were independent prognostic factors for poor OS (P=0.04 and P=0.03, respectively), while gross extrathyroidal extension, extrapulmonary distant metastases, and RR-WDTC were independent prognostic factors for poor DSS at the end of follow-up (P=0.02, P=0.03, and P=0.02, respectively).

**Conclusions:**

WDTC with distant metastases at initial diagnosis accounted for 31.15% of all elderly patients with DTC. Gross extrathyroidal extension and RR-DTC were the major factors associated with poor OS; gross extrathyroidal extension, extrapulmonary distant metastases, and RR-DTC were independent prognostic factors for poor DSS in elderly DTC patients with distant metastases.

## Introduction

Over the past two decades, differentiated thyroid cancer (DTC) has become the most common endocrine malignancy with the fastest growth rate worldwide (1). Despite its rising incidence, it has an excellent prognosis, and the vast majority of patients sustain long-term survival, with the 10-year overall survival (OS) rate currently >90% after total or near-total thyroidectomy combined with radioiodine (^131^I) ablation therapy (2). Previous studies have shown that age at initial diagnosis of DTC is the factor most associated with recurrence, survival, and prognosis (3). Elderly patients have a poorer prognosis and more aggressive DTC, which is characterized by larger tumors, extrathyroidal invasion, and distant metastasis (4). Although the AJCC/UICC revised the age cut-off from 45- to 55-years-old in the latest TNM staging system (2016), elderly DTC patients continue to have significantly decreased survival compared with younger patients (5). Regarding the optimal cut-off value for defining “the elderly” DTC population, 60, 65, and 70 years have been reported in different studies (6, 7). According to the World Health Organization, most developed countries have adopted the definition of ≥65 years as “the elderly”.

It has been reported that the prevalence of distant metastasis is relatively rare in DTC, with previous reports varying from 4% to 23% (8, 9). Lung and bone are the most common distant metastatic sites, and other less common organs include brain, kidney, skin, muscle, and pleura (10). For nearly eighty years, ^131^I has been the main treatment for distant DTC metastases after thyroidectomy. For radioiodine-refractory (RR)-DTC with distant metastases, clinical trials have demonstrated that molecular targeted drugs have good therapeutic efficacy, advancing the prospects for these patients. Now, sorafenib and lenvatinib are recommended as the first-line therapy for progressive RR-DTC according to the latest ATA management guidelines (11–13). It has been reported that several independent prognostic factors are related to the poor prognosis of DTC patients with distant metastases, such as age at initial diagnosis of distant metastases, loss of ^131^I avidity for distant metastases, presence of extrapulmonary distant metastases, and patients who initially present with distant metastases (8, 9, 14, 15).

Although the incidence of distant metastases in elderly DTC patients has significantly increased (4); to the best of our knowledge, there is little published information on the long-term outcomes of elderly well-DTC (WDTC) patients with distant metastases. To address this issue and raise awareness of this disease, this study reviewed the experience of a single tertiary medical center in China with a relatively large series of elderly (≥65 years) WDTC patients who presented with distant metastases at initial diagnosis. We analyzed the clinicopathological characteristics, long-term outcomes, and independent prognostic factors for overall survival (OS) and distant metastatic disease-specific survival (DSS) in these elderly patients with distant metastases.

## Materials and Methods

### Eligible Patients

**Figure 1:**
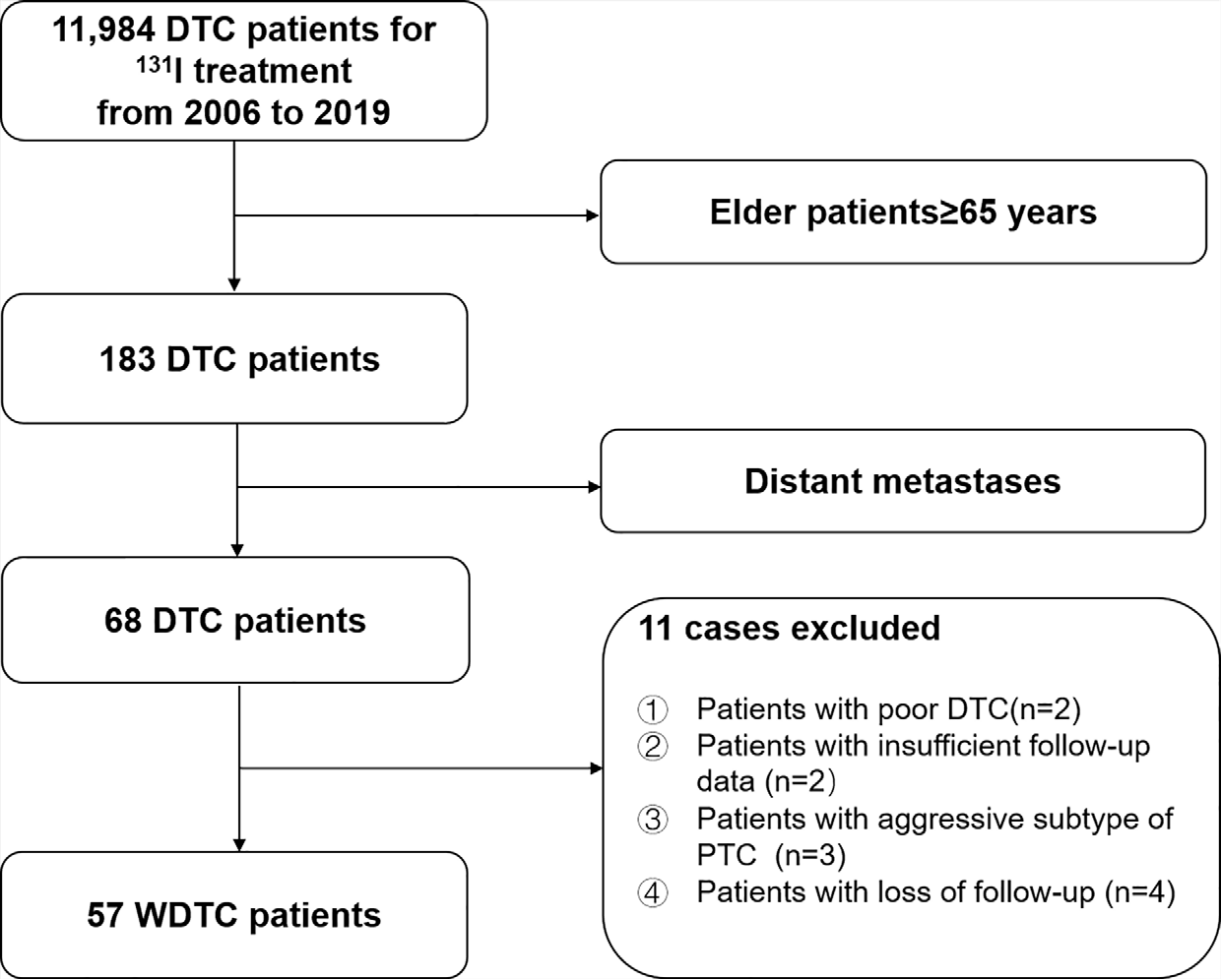
Flow diagram of the selection process for elderly (≥65-years-old) well-differentiated thyroid cancer (WDTC) patients with distant metastases at initial diagnosis.

We retrospectively reviewed the medical records of 11,984 consecutive DTC patients treated with ^131^I after total or near-total thyroidectomy between 2006 and 2019 at Shanghai Sixth People’s Hospital, a major ^131^I treatment center in China. In total, 57 elderly WDTC patients were enrolled in this study (**Figure 1**). This study was approved by the Institutional Review Board of the Shanghai Sixth People’s Hospital.

### Baseline Variables

Demographics, clinicopathological characteristics, ^131^I treatment results, and follow-up data for all eligible patients are shown in **Table 1**. Age was determined at the time of initial diagnosis of distant metastases from WDTC. Pathological type only included classic PTC and follicular thyroid cancer (FTC). Follicular variant of PTC (FV-PTC) and Hürthle cell cancer were classified into the PTC and FTC subtypes, respectively. Poor-DTC and aggressive subtypes of PTC were excluded. Extrathyroidal extension was divided into three categories: “No,” “Minimal,” and “Gross” according the 2015 ATA guidelines (11). “Minimal” was defined as microscopic tumor invasion into the perithyroidal soft tissues, while “Gross” was defined as macroscopic tumor invasion into perithyroidal soft tissues. N stage was evaluated based on the eighth edition of the AJCC/TNM cancer staging system (16). According to a past report, distant metastases confirmed within 1 year from the initial thyroid surgery were considered distant metastases at initial presentation, while distant metastases detected >1 year after initial diagnosis were considered delayed distant metastases (15).

**Table 1:** Patient characteristics of well-differentiated thyroid cancer (WDTC) with distant metastases.

	N (%) or Value
**Age at initial diagnosis of distant metastases (years) (Mean±SD, Median, Range)**	69.84±5.01,68,65–86
65–70	34(59.65%)
≥70	23(40.35%)
**Gender**	
Male	24(42.11%)
Female	33(57.89%)
**Number of thyroid surgeries**	
1	46(80.70%)
>1	11(19.30%)
**Pathological type**	
PTC	46(80.70%)
FTC	11(19.30%)
**Maximal primary tumor size (cm) (Mean ± SD, Median, Range)**	3.30±1.74,3.2,0.4–10.2
<2	10(17.54%)
2–4	34(59.65%)
≥4	13(22.81%)
**Tumor multifocality**	
No	36(63.16%)
Yes	21(36.84%)
**Extrathyroidal extension**	
No	33(57.90%)
Minimal	9(15.79%)
Gross	15(26.31%)
**N stage**	
N0	13(22.81%)
N1a	25(43.86%)
N1b	19(33.33%)
**Local persistence or recurrence during ^131^I treatment and follow-up**	
No	37(64.91%)
Yes	20(35.09%)
**Timing of distant metastasis**	
At initial presentation	12(21.05%)
Delayed distant metastases	45(78.95%)
**Symptom of distant metastases at initial diagnosis**	
Asymptomatic	30(52.63%)
Symptomatic	27(47.37%)
**Site of distant metastases at initial diagnosis**	
Lung only	33(57.89%)
Bone only	8(14.04%)
≥2 Organ system	16(28.07%)
**Treatment modalities of distant metastases**	
^131^I treatment	57(100.00%)
Palliative surgical treatment	4(0.07%)
External radiotherapy	7(12.28%)
TKIs therapy	8(14.04%)
**^131^I avidity**	
Yes	32(56.14%)
No	25(43.86%)
**RR-DTC**	
No	22(38.60%)
Yes	35(61.40%)
**Preablation stimulated Tg (ng/mL)(Mean±SD, Median, Range)**	498.32±11124.39,430.3,0.05–49320
**^131^I Cumulative administered(Mean±SD, Median, Range)**	15.58±14.94,11.10,3.7–72.5
**Number of courses for ^131^I therapy (Mean±SD, Median, Range)**	2.75±2.34,2.0,1–12

### Diagnosing Distant Metastases From WDTC

Diagnoses of distant metastases from WDTC were established and confirmed by at least one of the following criteria, which was similar to our previous study (17, 18). Criterion I: distant metastases were found or detected according to clinical manifestations and comprehensive imaging such as computed tomography (CT), magnetic resonance imaging (MRI), and/or ^18^F-fluorodeoxyglucose positron emission tomography/computed tomography (^18^F-FDG-PET/CT), and pathological results from operations or biopsies that confirmed the lesions originated from thyroid tissue; Criterion II: ^131^I uptake of distant metastatic lesions was found on therapeutic ^131^I whole body scan (^131^I-WBS), with the distant metastases detected on at least one imaging modality, including ^131^I-single-photon emission computed tomography/computed tomography (^131^I-SPECT/CT), CT, and/or MRI; and Criterion III: even if therapeutic ^131^I-WBS did not show positive lesions but the DTC patients had elevated thyroglobulin (Tg) or progressively increased thyroglobulin antibody (TgAb) levels, the distant metastases could be found on ^18^F-FDG-PET/CT scans.

### ^131^I Treatment of Distant Metastases

To implement ^131^I treatment after total or near-total thyroidectomy, all DTC patients consumed a low-iodine diet and were withdrawn from levothyroxine therapy for at least two weeks to achieve thyroid stimulating hormone (TSH) levels of ≥30 mIU/L, according to the ATA guideline (11). All patients were subjected to routine measurements of TSH, free triiodothyronine (FT3), free thyroxine (FT4), Tg, and TgAb, neck ultrasonography (US), CT, and/or MRI scans prior to ^131^I treatment. Next, if distant metastasis were diagnosed before ^131^I treatment, patients received an oral dose of ^131^I with standard activity of 5.55–7.40 GBq (150–200 mCi) to ablate remnant thyroid disease and treat distant metastases. If distant metastases were diagnosed on the ^131^I-WBS and another imaging modality after the first ^131^I therapy, 3.7 GBq (100 mCi) was used for the initial treatment to ablate remnant thyroid disease, and then 5.55–7.40 GBq (150–200 mCi) was taken orally for subsequent treatments aimed at treating distant metastases. The intervals for repeated ^131^I treatments were 4–12 months. Subsequent ^131^I treatments were interrupted in patients with weakly ^131^I-avid or non-^131^I-avid distant metastases on therapeutic ^131^I-WBS.

### Evaluating RR-DTC

We evaluated RR-DTC according to the 2015 ATA management guidelines, which defined structurally evident RR-DTC depending on the following criteria: (1) the primary and/or metastatic lesions do not absorb ^131^I on ^131^I-WBS; (2) the primary and/or metastatic lesions lose the ability to concentrate ^131^I after previously being ^131^I-avid lesions; (3) ^131^I is heterogeneously concentrated into some lesions on ^131^I-WBS but not others; and (4) primary and/or metastatic lesions show progressive disease (PD) within 1 year following ^131^I treatment despite sustained concentration of ^131^I. The therapeutic effect of ^131^I was divided into two categories: PD and non-PD. Similar to our previous report (15), PD responses were evaluated by the Response Evaluation Criteria in Solid Tumors Guideline Version 1.1 (RECIST1.1) (19) for pulmonary metastases and the MDA criteria (20) for bone metastases. For pulmonary metastases from DTC, PD was defined as at least a 20% increase in the sum of diameters for all measured target lesions with an absolute increase of at least 5 mm in the sum of diameters and/or the appearance of new lesions, while for bone metastases from DTC, PD was defined as at least a 25% increase in the size of measurable lesions using CT or MRI, at least a 25% subjective increase in the size of ill-defined lesions using CT or MRI, and/or the appearance of new lesions.

### Other Treatments for Elderly Patients With Distant Metastases

For RR-DTC, molecular targeted therapies, such as tyrosine kinase inhibitors (TKIs), have emerged as new treatment modalities (21, 22). The ATA guidelines recommend sorafenib and lenvatinib for the treatment of progressive RR-DTC (11–13). Since September 2017, only sorafenib has been approved as a first-line drug for progressive RR-DTC in China. In this study, sorafenib was used to treat progressive RR-DTC with distant metastases in eight patients. Other treatment modalities for distant metastases include external radiotherapy, palliative surgery, chemotherapy, bisphosphonates, and interventional therapy (11). In addition to ^131^I therapy, palliative surgery was performed in four patients, and seven patients underwent external radiotherapy during ^131^I treatment and follow-up. No other therapies were performed on the elderly patients with distant metastases in this study.

### Follow-Up Strategy

After ^131^I treatment, routine follow-up was performed for every patient, and the follow-up period for this study was until the end of June 2019. During the follow-up period, FT3, FT4, TSH, Tg, and TgAb levels were measured and physical examination and US of the neck were performed every 3–6 months, chest CT was performed annually to compare changes in the pulmonary metastatic lesions with previous results. For bone metastases, at least one imaging examination (CT, MRI, or ^18^F-FDG-PET/CT) was conducted annually to evaluate the clinical course of metastatic lesions and confirm PD. At the end of follow-up, the OS of these patients was calculated according to the time from initial detection of distant metastatic lesions to end of follow-up or death from any cause, while DSS was calculated from the time of initially detecting distant metastatic lesions to the end of follow-up or death from the distant metastatic disease.

### Statistical Analysis

All statistical analyses were performed using GraphPad Prism 7 (GraphPad Software, San Diego, CA, USA) and MedCalc version 17.0 (MedCalc Software, Mariakerke, Belgium). Continuous variables are presented as median and means ± standard deviations with range. Categorical variables were expressed as exact numbers with proportions. OS and DSS survival curves were analyzed by Kaplan–Meier method from the initial diagnosis of distant metastasis to the end of follow-up or death. To evaluate prognostic factors for OS and DSS at the end of follow-up, univariate analysis was performed using the Log-rank test, and then multivariate analysis was calculated using the Cox proportional hazard model. A p-value <0.05 was considered significant. Parameters with P-values <0.1 that displayed significant influence on the OS and DSS rates of elderly WDTC patients with distant metastases in univariate analyses were included in multivariate analyses. In contrast, parameters with P-values ≥0.1 were not included in multivariate analyses.

## Results

### Patient Demographics

Between 2006 and 2019, 183 elderly patients (≥65-years-old) with DTC were treated with ^131^I after total or near-total thyroidectomy in our department. Among them, 57 (31.15%) elderly WDTC patients with distant metastases were confirmed according to the inclusion criteria. Baseline clinicopathological features of these elderly WDTC patients with distant metastases are shown in **Table 1**.

### Diagnoses and Clinical Symptoms of Distant Metastasis

According to the diagnostic criteria for distant metastases in elderly WDTC patients, 13 cases (22.81%) were diagnosed according to Criterion I, 29 (50.88%) were diagnosed by Criterion II, and 15 (26.32%) were detected by Criterion III. Among the cases detected by Criterion III, three were negative for Tg and had persistently elevated TgAb levels, while the remaining 12 cases with positive Tg levels were included. In this study, 27 (47.37%) patients were symptomatic and 30 (52.63%) lacked any clinical symptoms upon the initial diagnosis of distant metastases. Among the 27 patients with clinical symptoms, 15 suffered from cough and expectoration, fiver had hemoptysis, six suffered from shortness of breath and/or breathing difficulties, three cases had pleural effusion, nine hade bone pain, two had pathologic fractures, and one patient had spinal cord compression.

### The Distribution of Initial Distant Metastases

Among the 57 elderly DTC patients with distant metastases, 33 (57.89%) were found to have lung metastases alone, eight (14.04%) presented with only bone metastases, and 16 (28.07%) had at least two synchronous distant organ metastases including 10 with synchronous bone and lung metastases, two with synchronous lung and brain metastases, two with synchronous lung and renal metastases, one with synchronous bone and brain metastases, and one with synchronous bone, lung and renal metastases (**Table 1**). Among the 48 cases with lung metastases, 17 were classified as micro-metastases (<1 cm) and 31 as macro-metastases (≥1 cm) according the largest size of the lung lesions. For the 20 patients with bone metastases, 16 had multiple bone metastases and four had a single bone metastasis.

### Response to ^131^I Therapy

**Figure 2:**
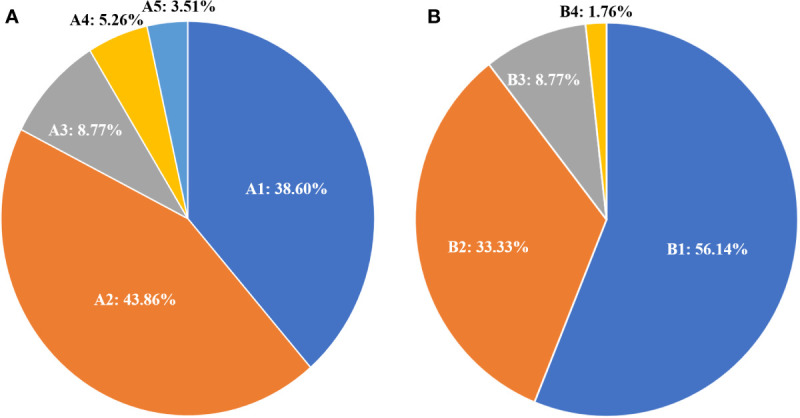
Distribution and percentage of ^131^I treatment status according to the RR-DTC evaluation criteria **(A)**; A1: non-RR-WDTC; A2: identified by Criterion I; A3: by Criterion IV; A4: by Criterion II; and A5: by Criterion III. Distribution and percentage of causes of death for these patients **(B)**; B1: patients alive at the last follow-up; B2: patients who died of distant metastases; B3: patients who died of other reasons; B4: the patient who died of locally invasive disease.

The responses to ^131^I treatment are showed in **Table 1**. ^131^I-WBS showed that 32 elderly patients (56.14%) could concentrate ^131^I, while 25 (43.86%) did not show therapeutic ^131^I concentration ability. According to the evaluation criteria for RR-DTC, 35 cases (61.40%) were classified as RR-WDTC and 22 (38.60%) as non-RR-WDTC. Among the 35 RR-DTC cases, 25 (43.84%) were identified by criterion 1, three (5.36%) by criterion 2, two (3.51%) by criterion 3, and five (8.77%) by criterion 4 (**Figure 2A**). According to the evaluation criteria of PD during ^131^I treatment, 40 patients (71.18%) did not show PD, while 17 (29.82%) had PD, including 12 who were classified by criterion 1, one by criterion 2, and five by criterion 4.

### Clinical Outcomes

**Figure 3:**
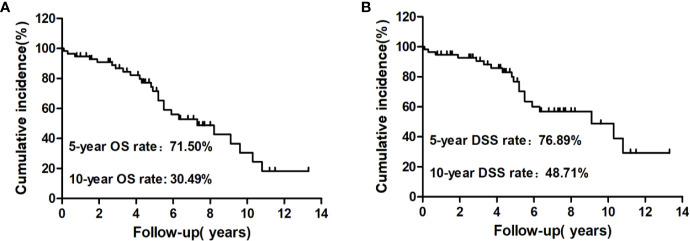
Kaplan–Meier analysis of cumulative overall survival (OS) **(A)** and disease-specific survival (DSS) **(B)** rates from the initial diagnosis of distant metastasis to the end of follow-up for these patients.

At the end of follow-up, 25 patients (43.86%) had died and 32 (56.14%) were alive. Among those who had died, 19 (33.33%) were caused by distant metastatic disease, including 10 from lung metastases, six from bone metastases, two from brain metastases, and one from renal metastasis; five (8.77%) deaths were caused by other causes, including one patient from heart failure, one from colorectal cancer, one from lung infection, two from cerebrovascular diseases, and one (1.76%) from locally invasive WDTC (**Figure 2B**). The median survival after the initial diagnosis of distant metastasis was 7.3 years (range: 0.1–13.3 years). The OS rate was 18.29% at the last follow-up, with 5- and 10-year OS rates of 71.50% and 30.49%, respectively (**Figure 3A**). The DSS rate was 29.23% at the last follow-up, with 5- and 10-year DSS rates of 76.89% and 48.71%, respectively (**Figure 3B**).

### Prognostic Factors for OS

**Figure 4:**
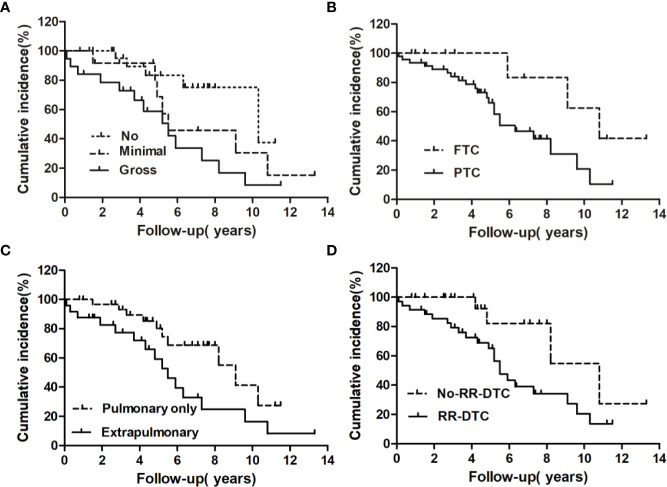
Univariate analysis of the prognostic factors for median overall survival (OS) at the end of follow-up; the median OS of patients differed significantly according to extrathyroidal extension **(A)**, pathological type **(B)**, site of distant metastases at initial diagnosis **(C)**, and RR-DTC status **(D)**.

The univariate analysis of prognostic factors for elderly DTC patients with distant metastases at the last follow-up is shown in **Table 2**. Four factors were found to be associated with OS, including pathological type, extrathyroidal extension, site of distant metastases at initial diagnosis, and RR-DTC status. Patient stratification showed that the median OS values of elderly patients with DTC who had gross extrathyroidal extension, extrapulmonary distant metastases, and RR-DTC were significantly lower than those of patients with FTC, no or minimal extrathyroidal extension, pulmonary only or no distant metastases, and non-RR-DTC (P=0.04, P=0.02, P=0.03, and P=0.02, respectively) (**Figure 4**). However, no significant differences were observed in OS for the other factors (P>0.05) (**Table 2**). Multivariate analyses that included five factors (P<0.1) from the univariate analysis indicated that gross extrathyroidal extension (hazard ratio [HR]: 3.07, 95% confidence interval [CI]: 1.413–9.382, P=0.04) and RR-DTC (HR: 2.36, 95% CI: 1.029–6.314, P=0.03) were independent factors associated with poor prognosis (**Table 3**). The other three factors from the univariate analysis (pathological type, N stage, and site of distant metastases) were not independent prognostic factors for OS (P>0.05) (**Table 3**).

**Table 2:** Univariate analysis showed the influence of prognostic factors on the overall survival (OS) of the elderly well-differentiated thyroid cancer (WDTC) patients with distant metastases by using the Log-rank test at the end of follow-up.

Variable	No. of patients	No. of deaths (%)	Log-rank	Hazard ratio	95 % Cl	P value
**Age at initial diagnosis of distant metastases (years)**			**2.43**			**0.12**
65–70	34	10 (29.41%)		1		
≥70	23	15 (65.22%)		1.67	0.742–3.760	
**Gender**			**1.46**			**0.23**
Male	24	8 (33.33%)		1		
Female	33	17 (51.52%)		1.63	0.738–3.615	
**Number of thyroid surgeries**			**0.07**			**0.80**
1	46	21(45.65%)		1		
>1	11	4 (36.36%)		0.88	0.313–2.447	
**Pathological type**			**4.13**			**0.04**
FTC	11	3(27.27%)		1		
PTC	46	22(47.83%)		2.61	1.035–6.556	
**Maximal primary tumor size**			**0.19**			**0.91**
<2	10	2(20.00%)		1		
2–4	34	15(44.12%)		1.17	0.282–4.894	
≥4	13	8(61.52%)		1.51	0.345–6.630	
**Tumor multifocality**			**0.64**			**0.43**
No	36	15(41.67%)		1		
Yes	21	10(47.62%)		1.12	0.500–2.495	
**Extrathyroidal extension**			**7.60**			**0.02**
No	26	5(19.23%)		1		
Minimal	12	7(58.33%)		2.13	0.654–6.952	
Gross	19	13(68.42%)		3.88	1.500–10.04	
**N stage**			**5.76**			**0.06**
N0	13	3 (23.08%)		1		
N1a	25	13 (52.00%)		2.470	0.753–8.070	
N1b	19	9 (43.37%)		4.405	1.508–12.860	
**Local persistence or recurrence**			**0.63**			**0.43**
No	37	16(43.24%)		1		
Yes	20	9(45.00%)		1.43	0.592–3.460	
**Timing of distant metastasis**			**0.64**			**0.42**
At initial presentation	12	5 (41.67%)		1		
Delayed distant metastases	45	20 (44.44%)		1.444	0.588–3.548	
**Symptom of distant metastases**			**1.70**			**0.19**
Asymptomatic	30	14 (46.67%)		1		
Symptomatic	27	11 (40.74%)		0.56	0.237–1.336	
**Site of distant metastases at initial diagnosis**			**4.85**			**0.03**
Pulmonary only	33	10 (30.30%)		1		
Extrapulmonary	24	15 (62.50%)		2.486	1.105–5.594	
**Treatment modalities of distant metastases**			**0.004**			**0.95**
RAI treatment	40	17 (42.50%)		1		
RAI combined with other treatment	17	8 (47.06%)		1.378	0.605–3.136	
**^131^I avidity**			**0.32**			**0.57**
Yes	32	14 (43.75%)		1		
No	25	11 (44.00%)		1.267	0.561–2.859	
**RR-DTC**			**5.16**			**0.02**
No	22	4 (18.18%)		1		
Yes	35	21 (60.00%)		2.57	1.138–5.800	

**Table 3:** Multivariate analysis of prognostic factors for the overall survival (OS) of the elderly differentiated thyroid cancer (DTC) patients presenting with distant metastasis according to Cox’s Proportional Hazards Model at the end of follow-up.

Variable	No. of patients	Hazard ratio	95 % Cl	P value
				
**Pathological type**				
FTC	11	1		
PTC	46	1.98	0.925–5.832	0.08
**Extrathyroidal extension**				
No	26	1		
Minimal	12	1.86	0.472–4.853	0.32
Gross	19	3.07	1.413–9.382	0.04
**N stage**				
N0	13	1		
N1a	25	1.320	0.553–4.162	0.27
N1b	19	1.871	0.708–10.354	0.22
**Site of distant metastases at initial diagnosis**				0.06
Pulmonary only	33	1		
Extrapulmonary	24	2.032	0.792–6.057	
**RR-DTC**				
No	22	1		
Yes	35	2.36	1.029–6.314	0.03

### Prognostic Factors for DSS

**Figure 5:**
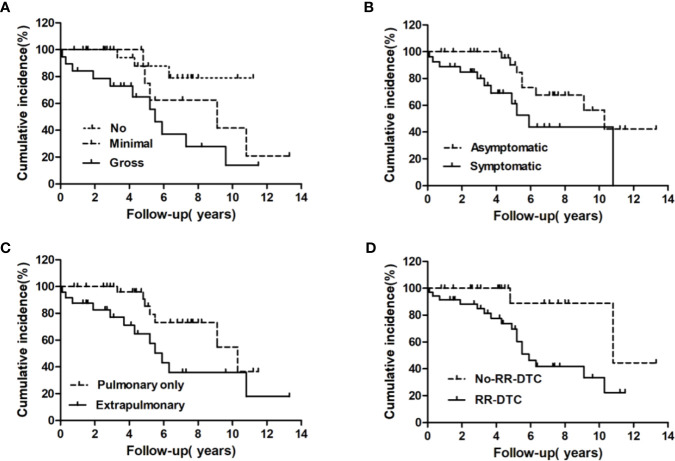
Univariate analysis of the prognostic factors for median DSS at the end of follow-up; the median disease-specific survival (DSS) of patients differed significantly according to extrathyroidal extension **(A)**, the presence of symptomatic metastases **(B)**, site of distant metastases at initial diagnosis **(C)**, and RR-DTC status **(D)**.

Factors predictive of DSS were determined by univariate analysis using the log-rank test (**Table 4**). Univariate analysis demonstrated that extrathyroidal extension, symptoms of distant metastases, site of distant metastases at initial diagnosis, and RR-DTC status were associated with DSS in these patients. Patients without or with minimal extrathyroidal extension, without symptoms of distant metastases, with only pulmonary metastases, and without RR-DTC had better median DSS rates those with gross extrathyroidal extension, symptomatic distant metastases, extrapulmonary distant metastases, and RR-DTC (P=0.01, P=0.02, P=0.02, and P=0.01, respectively) (**Figure 5**). Multivariate analysis that included four factors (P<0.1) from the univariate analysis showed that gross extrathyroidal extension (HR: 4.94, 95% CI: 1.511–17.319, P=0.02), extrapulmonary distant metastases (HR: 2.48, 95% CI: 1.030–9.001, P=0.03), and RR-DTC (HR: 2.89, 95% CI: 1.378–9.017, P=0.02) were independent prognostic factors related to poor DSS. There was no significant association between DSS and the presence of symptomatic distant metastases (P=0.05) (**Table 5**).

**Table 4:** Univariate analysis showed the influence of prognostic factors on the disease-specific survival (DSS) of the elderly well-differentiated thyroid cancer (WDTC) patients with distant metastases by using the Log-rank test at the end of follow-up.

Variable	No. of patients	No. of deaths (%)	Log-rank	Hazard ratio	95 % Cl	P value
**Age at initial diagnosis of distant metastases (years)**			**1.12**			**0.29**
65–70	34	8 (23.53%)		1		
≥70	23	11 (47.83%)		1.65	0.653–4.173	
**Gender**			**1.30**			**0.25**
Male	24	6(25.00%)		1		
Female	33	13(39.39%)		1.699	0.683–4.223	
**Number of thyroid surgeries**			**0.07**			**0.79**
1	46	16(34.78%)		1		
>1	11	3(27.27%)		0.85	0.263–2.774	
**Pathological type**			**1.85**			**0.17**
FTC	11	3(27.27%)		1		
PTC	46	16(34.78%)		2.08	0.721–6.098	
**Maximal primary tumor size**			**0.11**			**0.95**
<2	10	2(20.00%)		1		
2–4	34	11(32.35%)		1.16	0.229–5.848	
≥4	13	6(46.15%)		0.77	0.159–3.759	
**Tumor multifocality**			**0.17**			**0.68**
No	36	10(27.78%)		1		
Yes	21	9(42.86%)		1.121	0.481–3.048	
**Extrathyroidal extension**			**8.86**			**0.01**
No	26	3(11.54%)		1		
Minimal	12	5(41.67%)		2.37	0.558–10.08	
Gross	19	11(57.89%)		5.31	1.806–15.61	
**N stage**			**3.82**			**0.15**
N0	13	3(23.07%)		1		
N1a	25	10(40.00%)		3.26	0.993–10.73	
N1b	19	6(31.58%)		1.83	0.464–7.222	
**Local persistence or recurrence**			**1.55**			**0.21**
No	37	11(29.73%)		1		
Yes	20	8(40.00%)		1.90	0.691–5.219	
**Timing of distant metastasis**			**0.33**			**0.56**
At initial presentation	12	4(33.33%)		1		
Delayed distant metastases	45	15(33.33%)		0.74	0.262–2.076	
**Symptom of distant metastases**			**5.23**			**0.02**
Asymptomatic	30	8(26.67%)		1		
Symptomatic	27	11(40.74%)		3.12	2.177–8.256	
**Site of distant metastases at initial diagnosis**			**5.08**			**0.02**
Pulmonary only	33	7(21.21%)		1		
Extrapulmonary	24	12(50.00%)		2.92	1.150–7.435	
**Treatment modalities of distant metastases**			**0.05**			**0.83**
RAI treatment	40	12(30.00%)		1		
RAI combined with other treatment	17	7(41.18%)		1.11	0.431–2.870	
**^131^I avidity**			**0.09**			**0.76**
Yes	32	11(34.38%)		1		
No	25	8(32.00%)		1.16	0.454–2.936	
**RR-DTC**			**6.24**			**0.01**
No	22	2(9.09%)		1		
Yes	35	17(48.57%)		3.29	1.293–8.383	

**Table 5:** Multivariate analysis of prognostic factors for the disease-specific survival (DSS) of the elderly differentiated thyroid cancer (DTC) patients presenting with distant metastasis according to Cox’s Proportional Hazards Model at the end of follow-up.

Variable	No. of patients	Hazard ratio	95 % Cl	P value
**Extrathyroidal extension**				
No	26	1		
Minimal	12	2.01	0.528–10.381	0.13
Gross	19	4.94	1.511–17.319	0.02
**Symptom of distant metastases**				
Asymptomatic	30	1		**0.05**
Symptomatic	27	2.02	0.992–7.256	
**Site of distant metastases at initial diagnosis**				
Pulmonary only	33	1		0.03
Extrapulmonary	24	2.48	1.030–9.001	
**RR-DTC**				
No	22	1		0.02
Yes	35	2.89	1.378–9.017	

## Discussion

Although the prevalence of distant metastasis from DTC in elderly patients has been increasing, there have been few reports about the incidence, clinicopathological features, long-term outcomes, and prognostic factors of patients over 65-years-old with distant metastases from WDTC. To the best of our knowledge, only Han et al. (23) have described this patient population, finding that 17 of 86 elderly patients (>65-years-old) experienced distant metastases from DTC between 1994 and 2012, with an incidence rate of 19.77%. In this study, we retrospectively found that 57 of 183 elderly WDTC patients had distant metastases, an incidence rate of 31.15%, which represents a relatively large series of elderly patients with distant metastases who were treated with ^131^I at a single center. Most studies have shown that distant metastases occurred in 4%–23% of DTC patients (8, 14, 24, 25), which was lower than the results of our study. Our data support the conclusion that elderly DTC patients are more prone to having distant metastases.

In previous studies, the 5-year OS rates of DTC patients with distant metastases ranged from 42% to 88% and the 10-year OS rates ranged from 25% to 77% (8, 24–34); the 5-year DSS rates of DTC patients with distant metastases have ranged from 57.9% to 80% and the 10-year DSS rates have ranged from 26% to 48.3% (27, 30, 33, 34). In our study, the 5- and 10-year OS rates were 71.50% and 30.49%, respectively, while the 5- and 10-year DSS rates were 76.89% and 48.71%, respectively. All of these studies, including ours, have found heterogeneous clinical outcomes and a wide range of mortality risks for DTC patients with distant metastases, which may be caused by different sample sizes, diagnostic criteria, inclusion and/or exclusion criteria, and treatment modes. For instance, our inclusion criteria only included the well-differentiated pathological type among all DTC patients ≥65 years with metastatic disease. Regarding DTC patients with distant metastases, previous studies have demonstrated several independent prognostic factors associated with poor prognosis, including age at initial diagnosis of distant metastases, ^131^I avidity, timing of distant metastases, pathological type, and presence of extrapulmonary distant metastases (8, 9, 14, 15). Because of the inconsistencies in these previous studies regarding OS and DSS, we thought it valuable to assess the prognostic factors in our cohort of elderly DTC patients with distant metastases. Our multivariate analysis revealed that gross extrathyroidal extension and RR-DTC were independent factors associated with poor OS, while gross extrathyroidal extension, extrapulmonary distant metastases, and RR-DTC were independent prognostic factors related to poor DSS in elderly WDTC patients with distant metastases.

The most common pathological types of of DTC include PTC and FTC. Previous studies have showed the different ratios between PTC and FTC of DTC patients with distant metastases, with ratios varying from 4:1 to 1:1 (8, 24–34). However, our study found a significantly higher ratio than any previous study, with a PTC to FTC ratio of 4.18:1. This difference may be due to the different pathological subtypes included in PTC and FTC. In our study, PTC consisted of classical PTC and its subtype FVPTC, while FTC comprised classical FTC and its subtype Hürthle cell cancer. Most previous studies have also found no significant differences in the prognoses of DTC patients with distant metastases between the PTC and FTC subgroups, which was in accordance with our findings (8, 14, 24–26, 28, 29, 31, 32). Although our study found that FTC had a better OS than PTC by univariate analysis, there was not a significant difference in OS between PTC and FTC by multivariate analysis.

Distant metastases from DTC are usually located in the lungs and/or bones. The sites of distant metastases can be divided into three categories: lung only, bone only, and lung and/or bone with or without other distant metastases (≥2 organ systems). All previous studies have found that lung-only metastasis accounted for the largest proportion of all distant metastases, with rates varying from 43% to 66%; these data are in concordance with our study (57.89%) (8, 24, 26–34). Most studies have found that the proportion of patients with bone-only metastases was significantly higher than that of patients with ≥2 distant organ system metastases (14, 24, 26, 28, 34). In contrast, a few studies have indicated that the proportion of cases with ≥2 organ system metastases was significantly higher than that of bone-only metastases cases (30, 32), which is what we found in this study (28.07% vs. 14.4%). This finding may be due to the fact that elderly patients are more likely to have multiple distant metastases. Most previous studies have found that patients with lung or bone metastases only had better survival outcomes than patients with synchronous bone and lung metastases (10, 17). However, in this study, the numbers of patients with bone-only metastases were relatively small, so this could have been caused by statistical deviation. Thus, we divided our patients into two groups: those with pulmonary-only and extrapulmonary distant metastases. Univariate analysis showed that there were significant differences between the two groups in OS, but multivariate analysis revealed that pulmonary-only metastasis was not an independent factor associated with poor OS. These differences may have been caused by the six patients who died from other causes, which may have led to biases. For example, univariate and multivariate analysis for DSS demonstrated that site of metastasis was a significant prognostic factor in these patients.

A previous meta-analysis demonstrated that gross extrathyroidal extension is a major predictive factor for distant metastases from DTC, with a prevalence rate in DTC patients with gross extrathyroidal extension of 14.3% compared with only 3.8% in DTC patients without gross extrathyroidal extension (35). However, it is unclear if gross extrathyroidal extension is a major prognostic factor for DTC patients with distant metastases because extrathyroidal extension has not been included as a variable for univariate or multivariate analyses in most previous studies (8, 14, 24, 26–28, 30–34). Additionally, the study by Hirsch et al. revealed that gross extrathyroidal extension was not associated with improved OS or disease progression in DTC (including PTC and FTC) patients with distant metastases (9). In another study, gross extrathyroidal extension was found to be associated with decreased cancer-specific survival in PTC patients with distant metastases (36). These unexpected results may be explained by statistical deviations from the relative frequency of gross extrathyroidal extension in FTC (37). In this study, we found that gross extrathyroidal extension was an independent prognostic factor for elderly DTC patients with distant metastasis upon initial diagnosis that was associated with poor OS and DSS. An explanation for this finding may be that elderly patients with gross extrathyroidal extension tend to show more dedifferentiation, which can increase the formation of metastatic lesions (38).

In previous reports, the incidence of non-^131^I-avid distant metastases in DTC patients was approximately 30% (14, 25, 30, 31, 39, 40). In this study, distant metastatic lesions did not accumulate ^131^I in 43.86% (25/57) of the patients, which is higher than previous reports. This could suggest that distant metastatic lesions are less likely to have ^131^I uptake in elderly (>65-years-old) patients than in those <45-years-old (41). However, this finding could also represent differences caused by selection bias, small sample sizes, and/or screening methods for the referral population in our department and/or in clinical management measures after DTC diagnosis. Any of these factors could also affect the incidence of non- ^131^I uptake in elderly DTC patients with distant metastases. ^131^I avidity is the strongest prognostic factor for survival in DTC patients with distant metastases. Some studies have indicated that ^131^I avidity was associated with longer OS, DSS, progression-free survival, and disease-free survival compared cases without ^131^I avidity (14, 25, 30, 31, 39, 40). However, we found no difference in the median OS or DSS between ^131^I avid and non-^131^I avid cases. A possible explanation for this finding is that in some elderly DTC patients, although the metastatic lesions could absorb ^131^I, they had insufficient ^131^I uptake or resistance to the effects of ^131^I therapy, so disease progression was found during ^131^I treatment and/or follow-up. The classification of RR-DTC has been widely used in clinical practice recently. A literature report showed that approximately 50% of DTC patients with distant metastases were evaluated as RR-DTC (42), which has an important impact on survival data, as the mean survival time of these patients is approximately 3–5 years and their 10-year OS rate is usually <10% (11). In our study, 35 (61.40%) elderly patients with distant metastases were estimated to be RR-DTC using the univariate and multivariate analyses, which was higher than previous reports and suggested that elderly WDTC patients with distant metasets had higher levels of dedifferentiation and poorer prognosis. Our study also revealed that RR-DTC was an independent factor for poor OS and DSS.

In addition to ^131^I treatment, other treatment modalities are usually used for RR-DTC with distant metastases, such as external radiotherapy, palliative surgery, chemotherapy, bisphosphonates, and interventional therapy. Currently, there is no curative treatment modality available except for complete surgical resection, which is not always possible. Generally, RR-DTC is not sensitive to external radiotherapy or chemotherapy. Although TKIs such as sorafenib and lenvatinib have been recommend for RR-DTC by the ATA management guidelines (11), the challenge of using these drugs is how to maintain their continued use and control their adverse effects; moreover, many clinical trials have shown that TKI treatment can only improve the PFS and objective response rate, but has limited efficacy in prolonging OS (21, 22). In this study, ^131^I combined with other treatment modalities were performed in 29.8% (17/57) of the elderly patients with distant metastases. When we compared outcomes with patients who only received ^131^I treatment, there was no evidence that ^131^I combined with other treatments improved the median OS or DSS of these elderly patients with distant metastases.

The primary limitations of this study were as follows: first, it has certain inherent limitations related to its retrospective observational nature; nonetheless, this is the first study to evaluate the clinical outcomes of elderly patients with distant metastases from WDTC. Second, as the study spanned a long period, selection bias may have occurred. Third, because this was a single-center study conducted in China, further studies are needed to confirm the repeatability of our results in other countries and with other races. Moreover, except for standardized ^131^I treatment, the individualized treatment model and incomplete acquisition of treatment data may have led to deviations in clinical outcomes.

In summary, to the best of our knowledge, this is the first study to evaluate the long-term outcomes and prognostic factors of distant metastases in elderly WDTC patients and included the largest series of patients treated with ^131^I at a single center to date. We found that the 5- and 10-year OS rates for these elderly patients with distant metastases from WDTC were 71.50% and 30.49%, respectively, while their 5- and 10-year DSS rates were 76.89% and 48.71%, respectively. Absence of or minimal extrathyroidal extension and non-RR-DTC were independent factors associated with improved OS, while absence of or minimal extrathyroidal extension, lung-only metastases, and non-RR-DTC were independent prognostic factors related to improved DSS in these patients. These findings may contribute to clinical decision-making during the treatment and follow-up of elderly DTC patients with distant metastases. However, the sample size should be further increased, and a future perspective study is needed to obtain more thorough conclusions.

## Data Availability Statement

The original contributions presented in the study are included in the article/supplementary material. Further inquiries can be directed to the corresponding authors.

## Ethics Statement

The studies involving human participants were reviewed and approved by The study protocol was approved by the Ethics Committee of the Shanghai Jiao Tong University Affiliated Sixth People’s Hospital. The patients/participants provided their written informed consent to participate in this study. Written informed consent was obtained from the individual(s) for the publication of any potentially identifiable images or data included in this article.

## Author Contributions

Z-LQ and Q-YL designed the study. Z-KS, C-TS, Z-LQ, and G-QZ conducted the statistical analysis. YW, H-JS, and CX collected the clinical data. Z-LQ wrote the whole paper. Z-LQ, Q-YL, and YW supervised and edited the paper. All authors contributed to the article and approved the submitted version.

## Funding

This work was supported by the National Natural Science Foundation of China (grant number 81771865 and 81901773), the Shanghai Key Discipline of Medical Imaging (grant number 2017ZZ02005), and retrospective research fund of the Shanghai Sixth People’s Hospital.

## Conflict of Interest

The authors declare that the research was conducted in the absence of any commercial or financial relationships that could be construed as a potential conflict of interest.
